# Editorial: Therapeutic Index for Nutraceuticals in Inflammation-Related Diseases: Efficacy, Bioavailability, Metabolism and Interactions With Drugs, Volume II

**DOI:** 10.3389/fphar.2022.868784

**Published:** 2022-03-23

**Authors:** Ilaria Peluso, Maura Palmery

**Affiliations:** ^1^ Research Centre for Food and Nutrition, Council for Agricultural Research and Economics (CREA-AN), Rome, Italy; ^2^ Department of Physiology and Pharmacology “Vittorio Erspamer”, Faculty of Pharmacy and Medicine, Sapienza University of Rome, Rome, Italy

**Keywords:** bioactive compounds, functional foods, medicinal plants, inflammation, oxidative stress, pharmacokenetics

## Introduction

This Frontiers Research Topic is part of a series (https://www.frontiersin.org/research-topics/8685/therapeutic-index-for-nutraceuticals-in-inflammation-related-diseases-efficacy-bioavailability-metab) that aims to collect preclinical (*in vitro* and *in vivo*) and human studies to evaluate the therapeutic index for natural occurring phytochemicals based on their molecular targets, metabolism, pharmacokinetic, and potential interactions with drugs, examining interindividual variations (redox, inflammatory, immune and healthy/disease status, genetic polymorphisms, microbiota diversity).

## Issue Content

This issue includes two research papers and two review articles. Concerning the problem of the low bioavailability of bioactive phytochemicals, Wang et al. studied the effects of Piperine (PIP) in rats, examining its artificial synthetic analog, silepcimide (ILE) on the pharmacokinetics of curcumin (CUR). In addition to PIP, ILE, and CUR, the authors evaluated the plasma concentration of the metabolite of CUR dihydrocurcumin (DHC). After combined administration of CUR and ILE, Cmax, T1/2, and the AUC (0-tn) of DHC increased, whereas Tmax decreased. Besides, results from experiments of intravenous injection/oral administration, and of reactions with small intestine homogenate without microbes of SD rats, suggest the involvement of the gastrointestinal microorganisms. The role of gut microbiota is described in the review of Yang et al., in the context of the effects of berberine (BBR) in atherosclerotic cardiovascular and metabolic diseases, in view of its low bioavailability. BBR modulates the composition of the gut microbiota and the gut microbe-dependent metabolites and reduces the inflammation induced by gut microbiota-derived lipopolysaccharide (LPS). The latter has been used in a Zebrafish model of inflammation in the work of Balkrishna et al., aimed at evaluating the anti-inflammatory effect of the Ayurvedic medicinal food, Patanjali Special Chyawanprash (PSCP). PSCP reduced the hyperventilation and the loss of physical activeness in zebrafish due to inflammation, as well as the expression levels of pro-inflammatory cytokines, including interleukin-6 (IL-6), tumor necrosis factor alpha (TNF-α), and interleukin-1 beta (IL-1β). Moreover, from the results in THP-1 cells, the authors suggested the involvement of the nuclear factor kappa-B (NF-κB). In the review of Anaeigoudari et al. the inhibition of NF-κB is among the mechanisms reported for Nigella sativa and catechin. In the “*Highlights*” of their work, the authors suggested that active compounds from Nigella sativa L., Camellia sinensis L., and Allium sativum L. showed antidiabetic, anti-inflammatory, antioxidant, lipolysis, hepatoprotective, and cardioprotective effects.

## Context and Futures Directions

The Severe Acute Respiratory Syndrome Coronavirus 2 (SARS-CoV-2) outbreak has led to an increase in investigations of the potential role of bioactive molecules from natural sources on human health and virus infections, as described in other Frontiers Research Topics (https://www.frontiersin.org/research-topics/14125/ethnopharmacological-responses-to-the-coronavirus-disease-2019-covid-19-pandemic; https://www.frontiersin.org/research-topics/16489/biomolecules-against-coronaviruses-molecular-aspects-multi-omics-and-systems-pharmacology; https://www.frontiersin.org/research-topics/14960/plant-products-for-antiviral-therapeutics; https://www.frontiersin.org/research-topics/17117/emerging-and-old-viral-diseases-antiviral-drug-discovery-from-medicinal-plants; https://www.frontiersin.org/research-topics/30920/emerging-and-old-viral-diseases-antiviral-drug-discovery-from-medicinal-plants-volume-ii; https://www.frontiersin.org/research-topics/19696/natural-products-as-potential-therapeutics-to-tackle-life-threatening-infections-from-field-to-marke). Although the antioxidant and anti-inflammatory effects of nutraceuticals may help to avoid the cytokine storms induced by the coronavirus disease 2019 (COVID-19), self-medication is a growing phenomenon (Haque et al.; Tekeba et al., 2021; Wegbom et al., 2021), as well as online medication purchasing (Jairoun et al., 2021; Fincham 2021). In the context of possible interventions against misinformation (including nutraceuticals, vitamins, and immune boosters) and the abuse of over-the-counter (OTC) medicines (Al Mazrouei et al., 2021; Marwitz 2021), the potential interactions between nutraceuticals and drugs require more attention. OTC are freely sold at pharmacies, parapharmacies, and supermarkets and Bertuccioli et al. (2022) recently reviewed the potential nutraceutical-drugs interactions of many botanicals, including case reports. The major limitation of the present issue is that it does not include this topic, despite it being one of the aims of the Research Topic.
